# Advancements in Pulmonary Embolism Management: Current Approaches and Future Directions

**DOI:** 10.31083/RCM44614

**Published:** 2025-11-21

**Authors:** Carlo Banfi, Carolina Montonati, Alfonso Ielasi, Benjamin Assouline, Maurizio Tespili, Karim Bendjelid, Raphaël Giraud

**Affiliations:** ^1^Department of Cardiothoracic Surgery, IRCCS Ospedale Galeazzi-Sant'Ambrogio, University of Milan, 20157 Milan, Italy; ^2^Geneva Hemodynamic Research Group, University of Geneva, 1211 Geneva, Switzerland; ^3^Département de Cardiologie, Institut Mutualiste Montsouris, 75014 Paris, France; ^4^U.O. Cardiologia Ospedaliera, IRCCS Ospedale Galeazzi Sant'Ambrogio, 20157 Milan, Italy; ^5^Intensive Care Unit, Geneva University Hospitals, 1205 Geneva, Switzerland

**Keywords:** pulmonary embolism, systemic thrombolysis, catheter-directed therapy, anticoagulants, ECMO

## Abstract

Pulmonary embolism (PE) is one of the leading causes of cardiovascular mortality, with a high 30-day mortality rate. Despite clear treatment guidelines based on patient risk profiles, evidence suggests a discrepancy between clinical practice worldwide and the recommendations outlined in these guidelines. This deviation is often due to the comorbidities present in patients with PE, which complicate management decisions. As a result, alongside traditional standard-of-care treatments, novel emerging therapies are being explored to address these challenges. This review aims to provide an overview of the current epidemiology, initial assessment strategies, conventional treatment options, and emerging therapeutic approaches for PE.

## 1. Introduction

Pulmonary embolism (PE) is currently the third leading cause of cardiovascular death [1, 2, 3]. Over the last several decades, despite the rising incidence of PE, mainly driven by the aging population and the increasing prevalence of cancer, the overall 30-day mortality has remained stable at around 16%, ranging from 9% to 44% [1, 4, 5]. Beyond the commonly acknowledged risk factors of aging and cancer, epidemiological studies have highlighted several additional contributors to the rising incidence of PE, including an increasing proportion of postoperative patients, prolonged immobilization, obesity, and long-distance travel. Indeed, approximately one-third of PE patients have a history of recent surgery, with particularly higher incidence following major interventions (e.g., 0.7–30% after orthopedic surgery), and the risk remains elevated for at least 12 weeks postoperatively across all types of surgery [6, 7]. Consequently, improved postoperative survival contributes to the rising incidence of PE. Moreover, the increasing prevalence of obesity—a well-established risk factor for venous thromboembolism (VTE)—further contributes to the growing incidence of PE [8]. Age-standardized prevalence of overweight and obesity is projected to increase by 30.7% globally over the next 30 years, highlighting the potential for a further rise in PE incidence in the coming decades [9].

According to current guidelines, the most strongly recommended therapeutic strategies range from anticoagulation to thrombolysis, depending on hemodynamic status and individual risk profile [10]. Nevertheless, a substantial proportion of patients with PE either have contraindications to these therapies or present high-risk clinical features, thereby warranting consideration of more intensive treatment approaches. Indeed, despite two meta-analyses of randomized controlled trials (RCTs) showing a significant reduction in early mortality in high-risk PE patients who underwent thrombolytic therapy, only 12–20% of these patients received the treatment, primarily due to contraindications [2, 11, 12, 13, 14]. This, together with the expected rise in PE incidence in the coming years, underscores the urgency of advancing therapeutic strategies. Therefore, this review aims to provide an overview of the current epidemiology, initial assessment strategies, and conventional treatment options, while also presenting the most recent therapeutic approaches supported by contemporary evidence, to outline a more up-to-date framework for the management of PE.

## 2. Epidemiology and Risk Factors

The annual incidence of PE ranges from 39 to 120 cases per 100,000 population [10, 13, 15, 16, 17]. Epidemiological studies have reported a rising incidence of PE over the past two decades [13, 15], primarily driven by an aging western population, the increasing prevalence of cancer, a growing proportion of postoperative patients, prolonged immobilization, obesity, and long-distance travel, as well as by improvements in the sensitivity of imaging techniques [2]. Instead, particularly after the fourth decade of life, the absolute incidence of PE increases, reaching 1 in 100 cases in older individuals [13, 18].

Ninety-six percent of patients treated for VTE, including deep vein thrombosis (DVT) and PE, have at least one recognized predisposing risk factor, and 76% have two or more [19]. These triggers may be transient or permanent, and distinguishing between them is of primary importance for the long-term management of anticoagulation. Strong provoking factors for VTE include lower-limb fractures, recent hospitalization for heart failure (HF), joint replacements, major trauma, recent myocardial infarction, previous VTE, and spinal cord injury [19, 20]. There are also other predisposing factors (e.g., cancer, infection, hormone replacement therapy, pregnancy, oral contraceptives, thrombophilia, high-altitude travel, prolonged immobility, obesity, or increasing age) that confer a moderate risk of VTE [19, 20]. Knowledge of predisposing conditions is crucial for assessing the clinical probability of PE, which is essential for timely recognition and early treatment [10]. However, in 40% of patients with PE, no predisposing factors are identified [21].

## 3. Clinical Presentation and Diagnosis 

The clinical presentation of PE encompasses a wide range of non-specific symptoms and signs, including dyspnea (either abrupt in onset or worsening), chest pain (due to pleural irritation or right ventricular (RV) ischemia), syncope (associated with RV dysfunction), hemoptysis, and hemodynamic instability [22, 23, 24, 25]. Hypoxemia and hypocapnia are also frequent findings in PE patients [26]. Furthermore, electrocardiographic findings indicative of right ventricular overload, along with radiographic signs such as oligemia, truncation of the hilar artery, and pulmonary consolidations, can strengthen the suspicion of PE [23]. Additionally, these two diagnostic methods can help promptly rule out other potential causes of dyspnea and chest pain. Finally, the identification of predisposing factors is of critical importance. In fact, the combination of symptoms, clinical findings, and predisposing factors for VTE allows for the assessment of the pre-test probability of PE, based on clinical judgment or by applying established prediction rules, such as the revised Geneva score and the Wells score [24, 27].

Assessing the clinical probability of PE is essential for the timely and appropriate initiation of anticoagulation, as recommended by current guidelines. Anticoagulation is recommended to be initiated without delay in patients with a high or intermediate clinical probability of PE who are not hemodynamically unstable, and with unfractionated heparin (UFH) in patients with suspected high-risk PE, while diagnostic workup is ongoing [10]. It is important to emphasize that the initiation of anticoagulant therapy is based solely on the pre-test clinical probability of pulmonary embolism, not on the diagnosis confirmed by subsequent diagnostic investigations.

To complete the diagnostic work-up, plasma D-dimer measurement is indicated to rule out PE in patients with low or intermediate clinical probability, due to its high negative predictive value [28]. However, it is not recommended in patients with high clinical probability, as normal results cannot reliably exclude PE [10, 29]. In patients with high clinical probability or a positive D-dimer test (using an age-adjusted cut-off: age × 10 mg/L for patients aged >50 years), computed tomography pulmonary angiography (CTPA) is indicated to confirm the diagnosis [10]. However, lower-limb compression ultrasonography (CUS) showing a positive result for proximal DVT is also sufficient to diagnose VTE (and PE) in patients with a clinical suspicion of PE [30]. In patients with suspected PE and hemodynamic instability, a transthoracic echocardiogram (TTE) is indicated to detect signs of RV dysfunction and/or overload [10]. This justifies emergency reperfusion treatment in patients with a high clinical probability of PE, when no other obvious causes for RV overload are identified, and immediate CTPA is not feasible [10, 31].

## 4. Risk Stratification and Guideline-Directed Treatments

Risk stratification of PE patients is a fundamental step in guiding appropriate treatment and identifying those who require advanced therapy. Initial stratification is based on the identification of symptoms and signs of hemodynamic instability, such as (a) cardiac arrest, (b) cardiogenic shock (systolic blood pressure (BP) <90 mmHg, or vasopressors required to achieve a BP ≥90 mmHg despite an adequate filling status, in combination with end-organ hypoperfusion), or (c) persistent hypotension (systolic BP <90 mmHg or a systolic BP drop ≥40 mmHg for >15 min, not caused by other causes) [10]. Patients with hemodynamic instability and confirmed PE by CTPA, or evidence of RV dysfunction/overload on TTE, are classified as high-risk for early mortality (≈12% of PE cases; in-hospital mortality 40–76.6%) [1, 13, 14, 32, 33]. According to guidelines, systemic thrombolysis (e.g., alteplase, streptokinase, and urokinase) is the recommended therapy in PE high-risk patients without contraindications, and anticoagulation with UFH must be initiated without delay (UFH administration is allowed during infusion of alteplase, but should be discontinued during infusion of streptokinase or urokinase) whilst the diagnostic workup is ongoing [10]. In patients with contraindications to thrombolysis or in those for whom thrombolysis fails, surgical pulmonary embolectomy is the first-line treatment indicated [10].

To stratify the risk of hemodynamically stable patients, further investigation is required based on clinical parameters according to the original Pulmonary Embolism Severity Index (PESI) or its simplified version (sPESI) (e.g., age, sex, cancer, chronic heart failure, chronic pulmonary disease, pulse rate, systolic blood pressure, respiratory rate, temperature, altered mental status, and arterial oxyhemoglobin saturation, RV dysfunction, and cardiac troponin levels) [10]. Indeed, RV dysfunction and elevated cardiac biomarkers have been shown to be strong predictors of early mortality in PE [34, 35, 36]. Combining PESI/sPESI, the presence of RV dysfunction, and elevated cardiac troponin allows classification of hemodynamically stable patients as follows: (a) intermediate-high risk (PESI ≥3/sPESI ≥1, RV dysfunction, and elevated troponin; ≈35% of PE cases); (b) intermediate-low risk (PESI ≥3/sPESI ≥1, or RV dysfunction, or elevated troponin; ≈34% of PE cases); and (c) low-risk (PESI <3/sPESI = 0, no RV dysfunction, and negative troponin; ≈19% of PE cases) [10].

For patients with intermediate- and low-risk PE, anticoagulation is recommended without delay. If initiated parenterally, low-molecular-weight heparin (LMWH) or fondaparinux is preferred over UFH. If initiated orally, a novel oral anticoagulant (NOAC) is recommended over vitamin K antagonists (VKAs) in the absence of contraindications [10].

## 5. Gaps in Real-World Practice

Although guideline-recommended classifications and corresponding treatment approaches exist, there are several grey areas when applying guidelines to real-world populations. First, even though systemic thrombolysis is the recommended treatment for high-risk patients, it is used in only 12–20% of high-risk PE patients [2, 13, 14]. This is due to contraindications found in nearly 40% of cases or the perceived too-high risk of bleeding based on clinical judgment [32]. In these patients, and in those for whom thrombolysis fails, surgical pulmonary embolectomy is the first-line treatment indicated. However, this procedure is associated with an in-hospital mortality rate ranging from 9.1% to 23.2% [32, 37]. Secondly, as shown in a post hoc analysis of the Pulmonary Embolism Thrombolysis (PEITHO) trial in patients with intermediate-high risk of PE, several clinical parameters—such as systolic blood pressure ≤110 mmHg, a respiratory rate >20/min, cancer, and chronic HF—are associated with worse prognosis [38]. Patients with these factors appear to benefit more from systemic thrombolysis than from anticoagulation, although thrombolysis is not indicated as first-line therapy according to current guidelines due to the elevated bleeding risk. Identifying other risk factors for higher mortality in patients with intermediate-high risk PE could help pinpoint those who may benefit from advanced therapies, such as new-emerging catheter-directed therapies (CDTs). Currently, these therapies should be considered for high-risk patients with contraindications to or failure of thrombolysis, and in other patients only after hemodynamic deterioration, which implies treating them at a more advanced stage of disease [10].

Although there is no formal recommendation for any specific scoring system to detect early decompensation in initially hemodynamically stable patients, some expert PE centers have started using the National Early Warning Score (NEWS) 2, which is based on respiratory rate, oxygen saturation, supplemental oxygen, systolic BP, heart rate, and level of consciousness [39]. In fact, this score has been shown to reliably predict 7-day intensive care unit admission and 30-day mortality in hemodynamically stable patients with confirmed PE [40]. Moreover, the NEWS 2 was also found to predict the composite endpoint of 30-day all-cause mortality and the need for advanced therapy, defined as systemic thrombolysis or CDTs. A NEWS ≥7 may identify patients who could benefit from advanced therapy [41]. In this subgroup, the 30-day all-cause mortality was 39% among those who did not receive advanced treatment, compared with 18% among those who did [41].

## 6. Supporting Therapies

Along with reperfusion therapy, in some cases, supportive therapies for respiration and/or hemodynamic are required.

Hypoxemia, due to the mismatch between ventilation and perfusion, is a common finding in severe PE. Oxygen supplementation is indicated when oxygen saturation is below 90%, and non-invasive ventilation (NIV) is preferred over intubation to avoid sedation and hypotensive drugs, when feasible [10]. Furthermore, invasive ventilation with positive-pressure ventilation, by reducing preload and increasing pulmonary vascular resistance, can worsen RV dysfunction and decrease cardiac output. In contrast, NIV provides a non-invasive approach that can improve oxygenation without the adverse hemodynamic effects associated with invasive positive-pressure ventilation, making it a preferred option for managing PE patients with RV dysfunction. Therefore, intubation should be performed only if the patient is unable to tolerate, cope with, or is non-responsive to NIV, or in cases of extreme instability (e.g., cardiac arrest). If intubation is required, anesthetic agents prone to causing hypotension should be avoided, and positive end-expiratory pressure (PEEP) should be applied with caution to minimize further RV compromise [10].

On the other hand, to support hemodynamics in cases of RV failure, a cautious fluid challenge (≤500 mL) can be used as an initial attempt to increase cardiac output, while closely monitoring central venous pressure to avoid volume overload [10].

Additionally, in patients with hemodynamic instability, the use of vasopressors and/or positive inotropes is often necessary. The drugs of choice are noradrenaline for vasopressor support and dobutamine for inotropic support [10].

In patients with high-risk PE presenting with circulatory collapse or cardiac arrest, temporary mechanical cardiopulmonary support, such as veno-arterial extracorporeal membrane oxygenation (VA-ECMO), has demonstrated clinical utility in maintaining systemic perfusion and oxygenation, thereby reducing the risk of irreversible neurological injury. VA-ECMO effectively unloads the acutely strained right atrium and ventricle, restores adequate end-organ perfusion pressure, and ensures sufficient gas exchange through oxygenation and carbon dioxide removal [42, 43].

In this context, VA-ECMO serves not only as a bridge to reperfusion—facilitating right ventricular recovery while thrombus burden is reduced via systemic or catheter-directed thrombolysis—but may also act as a stand-alone therapy in selected cases, functioning as a bridge to recovery when thrombolysis or thrombectomy is contraindicated or deemed unnecessary [44, 45, 46]. Moreover, ECMO is fully compatible with reperfusion strategies such as CDTs or surgical embolectomy and enables these interventions to be performed under hemodynamically stable conditions, further improving procedural safety and outcomes [47].

Furthermore, veno-venous ECMO (VV-ECMO) has also demonstrated its efficacy in patients with restored hemodynamics but persistent severe hypoxemia. Indeed, even after reperfusion, injury to the microcirculation can result in a reduction of the pulmonary vascular bed and an increase in RV afterload. VV-ECMO, by improving oxygenation and CO_2_ removal, has been shown to enhance RV function and reduce pulmonary artery resistance [48].

## 7. Anticoagulation

For patients with intermediate- and low-risk PE, anticoagulation is the first-line treatment and must be initiated without delay in those with high or intermediate clinical probability of PE, while the diagnostic workup is ongoing [10]. If initiated parenterally, LMWH or fondaparinux is preferred over UFH. If initiated orally, a NOAC is recommended over VKAs in the absence of contraindications such as severe renal impairment, during pregnancy and lactation, and in patients with antiphospholipid antibody syndrome [10]. Use of higher doses of apixaban for 7 days or rivaroxaban for 3 weeks has been demonstrated to provide non-inferior efficacy and a potentially improved benefit–risk profile compared with LMWH or VKA [49, 50]. A meta-analysis comparing VKA-treated versus NOAC-treated PE patients showed a significant reduction in the incidence of major bleeding, particularly at critical sites, with NOACs, while maintaining efficacy comparable to VKAs [51]. Regarding the recommended dosing regimens, apixaban and rivaroxaban allow treatment initiation with an initial higher dose (e.g., rivaroxaban 15 mg twice daily for 3 weeks followed by 20 mg once daily; apixaban 10 mg twice daily for 7 days followed by 5 mg twice daily). In contrast, edoxaban and dabigatran require at least 5 days of parenteral anticoagulation before switching to edoxaban (60 mg once daily or dabigatran 150 mg twice daily). With respect to dose adjustment in renal insufficiency, edoxaban requires a reduction to 30 mg once daily in patients with mild to moderate renal dysfunction (Creatinine Clearance (CrCl) 30–60 mL/min), as well as in those with a body weight <60 kg. Conversely, the dosages of dabigatran, rivaroxaban, and apixaban are not reduced within this range of CrCl. Use of apixaban in patients with CrCl <25 mL/min and of rivaroxaban, edoxaban, and dabigatran in patients with CrCl <30 mL/min is not recommended. There are fewer interactions when NOACs are given concomitantly with other drugs [10]. A special mention should be made regarding the choice of the appropriate anticoagulant regimen in patients with active cancer. In these patients, treatment with LMWH for 6 months should be considered over VKA, or alternatively, in patients without gastrointestinal cancer, edoxaban or rivaroxaban may be used [10]. The minimum duration of anticoagulation is 3 months for all patients, and at least 6 months for those with cancer. Extended anticoagulation is required in patients with recurrent VTE without transient/reversible risk factors, as well as in those without identifiable risk factors, with irreversible risk factors, or with ongoing active cancer [10].

Despite these recommendations being applicable to all intermediate-high risk patients indiscriminately, as mentioned above, there is evidence suggesting that patients with intermediate-high risk PE who present additional risk factors (e.g., systolic blood pressure ≤110 mmHg, respiratory rate >20/min, cancer, and chronic HF) could benefit from more advanced therapies, such as systemic thrombolysis [38]. Instead, up to 5.6% of intermediate-high risk PE patients experience hemodynamic decompensation and/or die within 72 hours of admission [52]. Therefore, careful monitoring of these patients is essential during the first 2–3 days after admission to promptly detect decompensation and determine eligibility for advanced treatment [10, 39]. However, while systemic thrombolysis may offer potential benefits over anticoagulation in intermediate-risk PE patients with high-risk features, it is essential to emphasize that fibrinolysis in this setting is associated with a significantly increased risk of major bleeding and stroke [38]. Therefore, systemic thrombolysis is not recommended in intermediate-risk PE.

Moreover, since almost one third of PE patients present with recent surgery, with a particularly higher incidence after major interventions (e.g., 0.7–30% following orthopedic surgery), the management of anticoagulant therapy in the perioperative setting deserves special attention, both for VTE prevention and PE treatment, given the intrinsically higher risk in this patient population [6]. From a preventive perspective, the first step is to assess both the patient’s individual VTE risk factors and the procedure-related risk. Based on this assessment, in the absence of additional risk factors and with low-risk interventions, anticoagulant therapy is not recommended; instead, only general thromboprophylaxis measures such as adequate hydration and early mobilization should be implemented. Conversely, in patients with additional risk factors or undergoing higher-risk surgery, pharmacological thromboprophylaxis with LMWH is indicated, provided there are no contraindications [53]. On the other side, if the postoperative course is complicated by low- or intermediate-risk PE, anticoagulant therapy should be initiated as soon as possible in patients with high or intermediate clinical probability of PE, while the diagnostic workup is still ongoing [10]. The initiation of anticoagulation must, however, be carefully balanced against the bleeding risk associated with the surgical procedure. According to current perioperative anticoagulation management guidelines, NOAC or LMWH bridging may be initiated 24 hours after low-bleeding-risk procedures and 48–72 hours after high-bleeding-risk procedures [54]. In cases where the bleeding risk remains high, or when earlier initiation of anticoagulation is required, UFH may be considered, given its short half-life, ease of monitoring, and the possibility of immediate reversal [10].

## 8. Systemic Thrombolysis

According to guidelines, systemic thrombolysis is the recommended therapy in high-risk PE patients without contraindications, associated with UFH infusion that must be initiated without delay at the time of suspicion of high-risk PE [10]. Furthermore, it is recommended as a rescue thrombolytic therapy in patients with hemodynamic deterioration on anticoagulation treatment [10]. The current regimen of systemic thrombolysis includes alteplase, streptokinase, and urokinase (Table 1). Of note, UFH administration is allowed during the infusion of alteplase, but should be discontinued during an infusion of streptokinase or urokinase [10]. This strength of recommendation is primarily based on two meta-analyses of RCTs that show a significant reduction in early mortality and PE recurrence compared to anticoagulation alone, despite a higher rate of major bleeding and fatal/intracranial hemorrhage (Table 2) [11, 12, 52, 55, 56]. Table 2 summarizes major RCTs and meta-analyses comparing thrombolytic therapy with anticoagulation in acute PE. It presents study design, population, treatment strategies, follow-up, and main outcomes. It is important to note that the studies included in the meta-analyses are not focused solely on high-risk PE, which represents only a small percentage of the population included, and that there is only one RCT, involving 8 patients, specifically addressing cardiogenic shock [11, 12, 56]. In hemodynamically unstable patients, the major benefits of thrombolysis have been observed when treatment is initiated within 48 hours of symptom onset and before overt hemodynamic collapse necessitating cardiopulmonary resuscitation (CPR) occurs, as preserved circulation is required to deliver the drugs effectively [13].

**Table 1.** **Thrombolytic regimens in pulmonary embolism**.

Thrombolytic agent	Generation	Bolus	Regimen	Heparin discontinuation
Alteplase	Second	10 mg over 1–2 min	90 mg over 2 h	NO
			Total of 1.5 mg/kg over 2 h*	
Streptokinase	First	250,000 IU over 30 min	100,000 IU/h over 12–24 h	YES
Urokinase	First	4400 IU/kg over 10 min	4400 IU/kg over 12–24 h	YES

*If patient weight <65 kg. h, hours; IU, international units; min, minutes.

**Table 2.** **Thrombolysis vs. anticoagulation in acute pulmonary embolism: evidence from key studies**.

Authors	Study design	Total population	Main inclusion criteria	Treatment	Control	Follow-up	Clinical endpoints
Year of publication							
Marti *et al*.	Meta-analysis of 15 RCTs	2057	Acute PE (HR PE included in only 3 studies)	Thrombolysis ± Heparin	Heparin Alone	In-hospital – 30 days	↓ early mortality (OR = 0.59, *p* = 0.034) and PE recurrence (OR = 0.50, *p* = 0.031)
2015 [11]				(N = 1033)	(N = 1024)		
							↑ major bleeding (OR = 2.91, *p * < 0.001) and fatal/intracranial haemorrhage (OR = 3.18, *p* = 0.008)
Chatterjee *et al*.	Meta-analysis of 16 RCTs	2115	Acute PE (HR PE 1.5%)	Thrombolysis ± Heparin	LMWH, VKA, fondaparinux, UFH	In-hospital – 30 days	↓ early mortality (OR = 0.53, *p* = 0.01)
2014 [12]				(including UACDT)	(N = 1054)		↑ major bleeding (OR = 2.71, *p * < 0.01)
				(N = 1061)			
Jerjes-Sanchez *et al*.	RCT	8	Cardiogenic shock PE related	Thrombolysis	Heparin Alone	2 years	↓ early mortality (0% vs 100%, *p* = 0.02)
1995 [56]				(N = 4)	(N = 4)		
Meyer *et al*.	RCT	1005	Acute PE intermediate high-risk	Thrombolysis ± Heparin	Heparin Alone	30 days after randomization	∼early mortality (OR = 0.65, *p* = 0.42) and PE recurrence (OR = 0.20, *p* = 0.12)
2014 [52]				(N = 506)	(N = 499)		
(PEITHO trial)							∼30-days mortality (OR = 0.73, *p* = 0.42)
							↑ major extracranial bleeding (OR = 5.55, *p * < 0.001)
							↑ stroke (OR = 12.1, *p* = 0.003)

HR, high-risk; IR, intermediate risk; LMWH, low-molecular-weight heparin; LR, low-risk; OR, odds ratio; PE, pulmonary embolism; RCT, randomized controlled trials; UACDT, ultrasound-assisted catheter-directed therapy; UFH, unfractionated heparin; VKA, vitamin K antagonist; PEITHO, Pulmonary Embolism Thrombolysis; ↓, reduction in; ↑, increase in; ∼, similar rate of.

Among intermediate-high risk patients in the PEITHO trial, systemic fibrinolysis was associated with a lower risk of hemodynamic decompensation, despite a higher risk of major bleeding and stroke, and a similar rate of early and 30-day mortality (Table 2) [52]. Therefore, routine systemic fibrinolysis in intermediate- or low-risk patients is contraindicated [10]. However, a post hoc analysis of the same trial has shown that patients with an intermediate-high risk of PE and presenting systolic BP ≤110 mmHg, a respiratory rate >20/min, cancer, and chronic HF appear to benefit more from systemic thrombolysis than from anticoagulation, although this was associated with a significantly increased risk of major bleeding and stroke [38].

Despite the survival benefit of thrombolysis in high-risk PE patients, several studies have reported its underuse in this population, with only 12–20% of high-risk PE patients receiving the treatment [2, 13, 14]. This is primarily due to contraindications encountered in nearly 40% of patients [2, 13, 14]. This underscores the need for alternative advanced treatments to address this high-risk population.

## 9. Surgical Embolectomy 

In high-risk PE patients with contraindications to systemic thrombolysis and in those for whom thrombolysis fails, surgical pulmonary embolectomy is the first-line treatment [10]. It should also be considered an alternative to rescue thrombolytic therapy in hemodynamically deteriorating low- and intermediate-risk PE patients on anticoagulation [10]. However, it has been used in 2.8% of high-risk PE patients and is associated with a high in-hospital mortality rate, ranging from 9.1% to 23.2% [14, 32, 37]. Indeed, centers performing surgical pulmonary embolectomy should possess not only surgical expertise but also strong capabilities in postoperative management, particularly for complications such as persistent RV dysfunction, cardiac tamponade, sternal wound infections, and postoperative bleeding. The two main risks associated with this surgical technique are the exacerbation of RV failure and systemic malperfusion. To mitigate these drawbacks, the use of cardiopulmonary bypass (CPB) during surgery has been shown to facilitate RV recovery by decompressing the dilated and dysfunctional RV through diversion of the cardiac output to a pump and oxygenator [32]. This reduces both preload and afterload, allowing RV contraction in a fully decompressed state, while CPB simultaneously supports systemic perfusion. Furthermore, avoiding aortic cross-clamping promotes RV recovery by preventing associated myocardial edema and dysfunction [32]. Furthermore, studies have shown a high risk of recurrent PE following surgery, which can be especially detrimental in patients with preexisting RV failure [57].

## 10. Catheter-Directed Therapy 

**Fig. 1.**
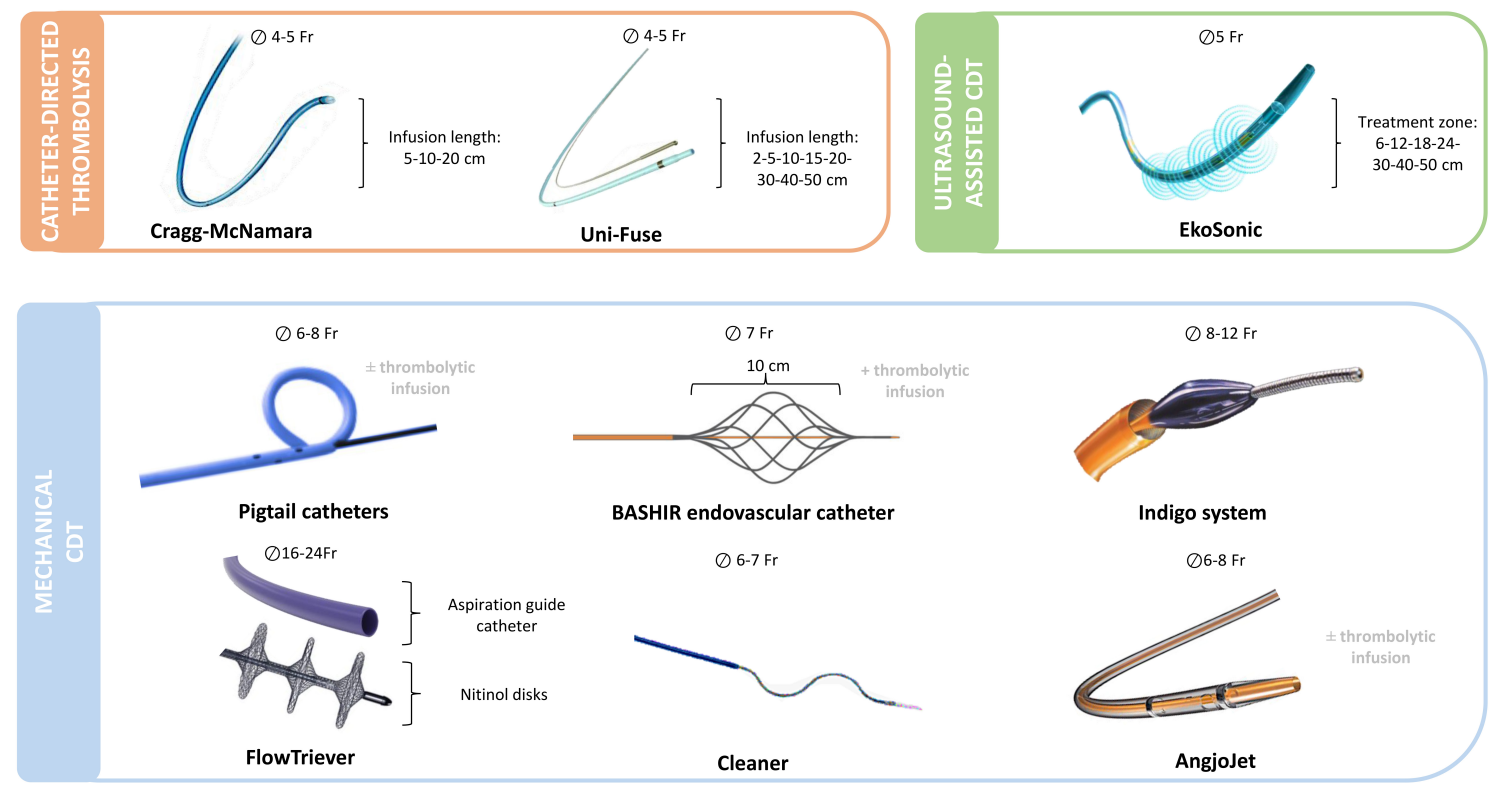
**Catheter-based treatments currently used for pulmonary embolism**. Overview of catheter-directed therapies grouped by mechanism of action: thrombolysis (orange), ultrasound-assisted thrombolysis (green), and mechanical thrombectomy (blue). Devices are shown with required venous access size (⊘), key structural features, and the possibility of adjunctive thrombolytic infusion [39, 59, 61, 62, 64, 65]. CDT, catheter-directed therapy; Fr, French.

In current guidelines, CDT should be considered for high-risk PE patients with contraindications to systemic thrombolysis, in those for whom thrombolysis fails, or as an alternative to rescue thrombolytic therapy in hemodynamically deteriorating low- and intermediate-risk PE patients on anticoagulation [10]. Despite the lack of strong recommendations in the guidelines due to the absence of large-scale randomized studies, CDT has been used in 25% of intermediate-risk patients and in 26% of high-risk patients [14]. CDT has shown a good safety profile, with a 30-day mortality rate of 0.9–2.7% and demonstrated favourable hemodynamic effects, including a reduction in the RV/left ventricle (LV) ratio and a decrease in systolic pulmonary artery pressure (sPAP) [58, 59, 60, 61, 62]. However, CDT may be associated with several adverse events, ranging from haemodynamic instability and respiratory failure to alveolar bleeding or even pulmonary artery perforation. Other reported complications include contrast-induced acute kidney injury with haemolysis, as well as haematomas at the vascular access site [39, 63]. The type and frequency of these adverse events largely depend on the specific technique and device employed. Nevertheless, these techniques have a rapid learning curve, and implementing measures such as echo-guided vascular access may help minimize the incidence of adverse events. CDT includes different systems, such as catheter-directed thrombolysis (CDTL), ultrasound-assisted CDT (UACDT), and mechanical thrombectomy (Fig. 1, Ref. [39, 59, 61, 62, 64, 65]). The main features of the current catheter-direct therapies for PE are described in Table 3 (Ref. [66]).

**Table 3.** **Main current catheter-direct therapies for pulmonary embolism (PE)**.

Device	Mechanism	Vascular access size (Fr)	CE approval for PE	Evidence in PE	Futures prospectives
Cragg-McNamara	Thrombolytic infusion	4–5	No	Kroupa *et al*. 2022 [66]	PE-TRACT Trial
(Medtronic)				CANARY Trial, SUNSET sPE Trial	
				PEERLESS	
Uni-Fuse	Thrombolytic infusion	4–5	No	SUNSET sPE Trial	PE-TRACT Trial
(Angiodynamics)				PEERLESS	BETULA
EkoSonic	Thrombolytic infusion + Ultrasound dispersion	5	Yes	SEATTLE II Study ULTIMA Trial	HI-PEITHO
(Boston Scientific)				OPTALYSE PE Trial	
				SUNSET sPE Trial	
				PEERLESS	
Pigtail catheters	Fragmentation ± Thrombolytic infusion	6–8	Not applicable	Case series	-
BASHIR endovascular catheter	Mechanical fragmentation + Aspiration + Thrombolytic infusion	7	No	RESCUE Study	HI-PEITHO
(Thrombolex)					
Indigo system	Mechanical fragmentation + Aspiration	8–12	Yes	EXTRACT-PE Trial	STRIKE-PE Study
(Penumbra)				STRIKE-PE Study interim analysis	STORM-PE
FlowTriever	Aspiration ± Mechanical fragmentation	16–24	Yes	FLARE Study	-
(Inari)				FLASH Study	
				FLAME Study	
				PEERLESS	
Cleaner	Mechanical fragmentation + Aspiration	6–7	No	Case series	CLEAN-PE
(Argon Medical)					
AngjoJet	Rheolytic thrombectomy + Aspiration ± Thrombolytic infusion	6–8	No	Case series	-
(Boston Scientific)					

BETULA, better efficacy and tolerability with ultrasound-assisted thrombolysis in acute pulmonary embolism; CANARY, catheter-directed thrombolysis in acute intermediate-risk pulmonary embolism study; CLEAN-PE, catheter-directed low-dose thrombolysis for acute pulmonary embolism; EXTRACT-PE, evaluating the penumbra indigo system for the treatment of pulmonary embolism; FLAME, flowtriever for acute massive pulmonary embolism; FLARE, flowtriever pulmonary embolectomy clinical study; FLASH, flowtriever all-comer safety and hemodynamics registry; HI-PEITHO, hybrid imaging and pulmonary embolism international thrombolysis study; OPTALYSE-PE, optimum duration of acoustic pulse thrombolysis procedure in acute pulmonary embolism; PEERLESS, pulmonary embolism evaluating the relative late effects of suction systems; PE-TRACT, pulmonary embolism thrombus removal with catheter-directed therapy; RESCUE, registry of the indigo aspiration system in acute pulmonary embolism; SEATTLE II, submassive and massive pulmonary thrombolysis therapy with ultrasound acceration II; STORM-PE, short-term outcomes after mechanical thrombectomy for pulmonary embolism; STRIKE-PE, study of rapid induction of kinetic energy for pulmonary embolism; SUNSET sPE, standard vs ultrasound-assisted catheter-directed thrombolysis in submassive pulmonary embolism; ULTIMA, ultrasound accelerated thrombolysis of pulmonary embolism; CE, European conformity; Fr, French.

### 10.1 Catheter-Directed Thrombolysis

The rationale for CDTL is based on the local delivery of thrombolytic agents inside the thrombus, maximizing the effect while lowering the total dose to reduce bleeding side effects. The Cragg-McNamara (Medtronic) and Uni-Fuse (AngioDynamics) catheters are the main devices available. The Cragg-McNamara catheter consists of two 4 French (Fr) catheters placed in the right and left interlobar pulmonary arteries via a single 8 Fr dual-lumen introducer, featuring a 10 cm infusion zone (Fig. 1, orange box) [66]. The Uni-Fuse catheter consists of a multi-hole catheter with an end hole and side hole placed in the clot for optimal intra-thrombus drug delivery (Fig. 1, orange box) [67]. The main characteristics of the principal studies concerning CDTL are described in Table 4 (Ref. [58, 59, 60, 61, 62, 64, 65, 66, 68, 69, 70, 71, 72, 73]).

**Table 4.** **Characteristics of the principal studies concerning catheter-directed therapies**.

Authors	Study design	Total population	Main Inclusion criteria	Treatment	Comparison	Follow-up	Clinical endpoints	RV	PAP
Year of publication									
Catheter-directed thrombolysis									
Kroupa *et al*. 2022 [66]	RCT	23	Intermediate-high risk PE	Cragg-McNamara with alteplase infusion + Heparin (N = 12)	LMWH/Heparin (N = 11)	30-days	-0% mortality at 30 days in both groups	↑ RV/LV ratio reduction of at least 25% (*p* = 0.03) in CDTL	↑ sPAP reduction of at least 30% (*p* = 0.001) in CDTL
							-No life-threatening bleeding reported in both groups		
Sadeghipour *et al*. 2022 [64] (CANARY Trial)	RCT	85	Intermediate-high risk PE	Cragg-McNamara with alteplase infusion + Heparin (N = 46)	LMWH/Heparin (N = 39)	3-month	-No differences in 3-month mortality	↓ median RV/LV at 72 hours (*p* = 0.01) and at 3-months (*p* = 0.001) in CDTL	∼30-months sPAP
							-No differences in bleeding		
Ultrasound-assisted CDT									
Piazza *et al*. 2015 [68] (SEATTLE II Study)	Single-arm, prospective, multicenter study	150	Intermediate risk PE	Ekos system and tPA + Heparin (N = 150)	-	30-days	30-day mortality = 2.7%	RV/LV diameter ratio reduction from 1.55 to 1.13 at 48 h (*p* < 0.0001)	sPAP reduction from 51 mmHg to 37 at 48 h (*p* < 0.0001)
							SAE related to device = 2%		
							30-day major bleeding = 10%		
Kucher *et al*. 2014 [65] (ULTIMA Trial)	RCT	59	Intermediate risk PE	Ekos system and tPA + UFH	UFH (N = 29)	90-days	No differences in 90-days mortality (*p* = 1) and bleeding (*p* = 0.61)	↑ RV/LV ratio reduction (*p* < 0.001) in CDTL at 24 h, with no differences at 90-days (*p* = 0.07)	↑ RV/RA gradient reduction (*p* = 0.03) in CDTL at 24 h, with no differences at 90-days (*p* = 0.91)
				(N = 30)					
Tapson *et al*. 2018 [69] (OPTALYSE PE Trial)	RCT	101	Intermediate risk PE	Ekos system and tPA at 4 mg/lung/2 h + Heparin	Ekos system and tPA at 4 mg/lung/4 h + Heparin	1-year	All-cause mortality = 2%	RV/LV diameter ratio reduction by 25% in all groups at 72 h (*p* < 0.01)	The refined modified Miller score, representing clot burden, improved as the dose increased and the infusion duration increased
				(N = 28)	(N = 27)		Major bleeding rate 3.6% at 72 h		
					Ekos system and tPA at 6 mg/lung/6 + Heparin				
					(N = 28)				
					Ekos system and tPA at 12 mg/lung/6 h + Heparin				
					(N = 18)				
Sterling *et al*. 2024 [70] (KNOCOUT PE)	Single-arm, prospective, multicenter study	489	Intermediate-high/high-risk PE	Ekos system and tPA + Heparin (N = 489)	-	12-month	30-day mortality = 1.6%	RV/LV diameter ratio reduction by 24.6% post-procedural (*p* < 0.0001)	Mean relative reduction of RV systolic pressure of 28.55% at 24–48 h (*p* < 0.001)
							30-day major bleeding = 1%	RV/LV diameter ratio reduction by 37.8% at 3-month (*p* < 0.0001)	
Mechanical CDT									
Bashir *et al*. 2022 [59] (RESCUE Study)	Single-arm, prospective, multicenter study	109	Intermediate-risk PE	BASHIR endovascular catheter	-	30-day	72-h major device-related AEs = 0.92%	RV/LV diameter ratio reduction by 33.3% at 48 h (*p* < 0.0001)	The refined modified Miller index reduction by 35.9% at 48 h (*p* < 0.0001)
				(N = 109)			72-h major bleeding = 0.92%		
							30-day mortality = 0.92%		
Sista *et al*. 2021 [61] (EXTRACT-PE Trial)	Single-arm, prospective, multicenter study	119	Intermediate-risk PE	Indigo system (N = 119)	-	30-day	48-h major device-related AEs = 0.8%	RV/LV diameter ratio reduction by 27.3% at 48 h (*p* < 0.0001)	sPAP reduction of 7.9%
							48-h major AEs = 1.7%		
							48-h major bleeding = 1.7%		
							30-day mortality = 2.5%		
Moriarty *et al*. 2024 [60] (STRIKE-PE Study, interim analysis)	Single-arm, prospective, multicenter study	150	Intermediate/high-risk PE	Indigo system (N = 150)	-	90-day	Major device-related AEs = 1.3%	RV/LV diameter ratio reduction by 25.7% at 48 h (*p* < 0.001)	sPAP reduction of 16.3% (*p* < 0.001)
							48-h major AEs = 2.7%		
							48-h major bleeding = 2.7%		
							30-day mortality = 2.0%		
Tu *et al*. 2019 [62] (FLARE Study)	Single-arm, prospective, multicenter study	109	Intermediate-risk PE	FlowTriever	-	48-h	Major device-related AEs = 0%	RV/LV diameter ratio reduction by 25.1% (*p* < 0.0001)	sPAP reduction (*p* < 0.001)
				(N = 109)			48-h major AEs = 3.8%		
							48-h major bleeding = 0.9%		
Toma *et al*. 2023 [58] (FLASH Registry)	Single-arm, prospective, multicenter study	250	Intermediate-/high-risk PE	FlowTriever	-	30-day	48-h major AEs = 1.2%	RV/LV diameter ratio reduction by 28.3% (*p* < 0.001)	sPAP reduction of 22.2% (*p* < 0.001)
				(N = 250)			30-day mortality = 0.4%		
Silver *et al*. 2023 [71] (FLAME Study)	Comparative, prospective, multicenter study	115	High-risk PE	FlowTriever	Other contemporary therapies: systemic thrombolysis (68.9%), anticoagulation (23%)	In-hospital	In-hospital mortality 1.9% vs 29.5%	-	-
				(N = 53)					
							Major bleeding 11.3% vs 24.6%		
							Bailout 3.8% vs 26.2%		
					(N = 61)				
CDT comparisons									
Avgerinos *et al*. 2021 [72] (SUNSET sPE Trial)	RCT	81	Intermediate-risk PE	Ekos system and tPA + Heparin	Cragg-McNamara or Uni-Fuse with tPA + Heparin	12-months	In-hospital death 1 in USAT vs 0 in CDTL	↑ RV/LV ratio reduction (*p* = 0.01) in CDTL at 48-h	No differences in thrombus burden reduction between the groups at 48-h
				(N = 40)	(N = 41)		Major bleeding 2 in USAT vs 0 in CDTL		
Jaber *et al*. 2025 [73] (PEERLESS)	RCT	550	Intermediate-risk PE	FlowTriever	Cragg-McNamara or Uni-Fuse or BASHIR endovascular catheter or Fountain (N = 276)	30-day	In-hospital mortality = 0% vs 0.4%, *p* = 1	-	-
				(N = 274)			Major bleeding = 6.9% vs 6.9%, *p* = 1		
							Clinical deterioration and/or bailout = 1.8% vs 5.4%, *p* = 0.04		
							30-day mortality 0.4% vs 0.8%, *p* = 0.62		

CDTL, Catheter-Directed Thrombolysis; SAE, Serious Adverse Event; AE, adverse event; LV, left ventricle; RV, right ventricle; sPAP, systolic pulmonary artery pressure; tPA, tissue plasminogen activator; USAT, ultrasound-assisted technology; ↓, reduction in; ↑, increase in; ∼, similar rate of.

RCTs conducted on intermediate-high risk populations have demonstrated a benefit of CDTL, using a Cragg-McNamara catheter, over anticoagulation alone in reducing the RV/LV ratio, with this effect even sustained over 3 months [64, 66]. A significant reduction in sPAP was also observed in CDTL-treated patients compared to the anticoagulation arm [66]. However, the clinical relevance of this hemodynamic improvement remains unclear, as these studies lacked sufficient power to evaluate potential effects on clinical endpoints such as all-cause mortality and bleeding. Interestingly, a network meta-analysis of 44 studies involving 20,006 intermediate-/high-risk patients showed that CDTL (also including ultrasound-assisted CDT) is associated with a reduced risk of death compared to both systemic thrombolysis (odds ratio (OR) = 0.43, *p* < 0.001) and anticoagulation (OR = 0.36, *p* < 0.001), as well as a decreased risk of major bleeding (OR = 0.61, *p* < 0.001) compared to systemic thrombolysis. Furthermore, no significant differences in major bleeding were found between CDTL and anticoagulation (OR = 1.24, *p* = 0.8) [74]. More definitive answers could be provided by two ongoing RCTs involving CDTL. The BETULA RCT (NCT03854266) includes intermediate-high risk PE patients treated with the Uni-Fuse system or heparin alone. Meanwhile, the Pulmonary Embolism Thrombus Removal With Catheter Directed Therapy (PE-TRACT) trial (NCT05591118) compares CDTL or mechanical thrombectomy plus anticoagulation versus anticoagulation alone in intermediate-high risk PE.

### 10.2 Ultrasound-Assisted CDT

Ultrasound-assisted technology works by generating an acoustic field that disperses the fibrinolytic agent into the clot and disaggregates the thrombus, separating the fibrin strands. This process aims to maximize the thrombus surface area and accelerate clot lysis [67]. The Ekos system (Boston Scientific) is the UACDT utilized in PE and consists of a 5-Fr infusion catheter and an ultrasound core transducer (Fig. 1, green box) [67]. The main characteristics of the principal studies concerning UACDT are described in Table 4.

This technology has shown excellent hemodynamic improvement in PE patients, demonstrating a reduction in the RV/LV ratio and sPAP, along with a good safety profile, as seen in the SEATTLE II trial reporting a 30-day mortality of 2.7% and a major bleeding rate of 10% in an intermediate-risk PE population [68]. These hemodynamic improvements have also been confirmed in a larger prospective registry, the KNOCOUT PE study, as well as in two RCTs [65, 70, 72]. The ULTIMA RCT, which enrolled 59 intermediate-risk patients randomly assigned to UACDT with recombinant tissue plasminogen activator (rtPA) plus UFH or UFH alone, demonstrated significantly greater early hemodynamic improvement in the UACDT group, in terms of reduction of the RV/LV ratio and right atrium (RA)/RV ratio (a surrogate for sPAP). However, at 90 days, both groups showed similar hemodynamic improvements. No significant differences were observed in terms of mortality and bleeding, although the small sample size limits the power to assess hard clinical endpoints [65]. Compared to standard CDTL, as analyzed in the SUNSET sPE RCT enrolling 81 intermediate-risk patients, UACDT showed a greater reduction in the RV/LV ratio. Both treatments demonstrated a reduction in thrombus burden; however, UACDT did not show a significantly greater improvement in thrombus load reduction compared to standard CDTL [72]. Regarding the dose of tPA infused via UACDT, there is no standard regimen. However, even a low dose has been shown to be effective in early RV/LV ratio reduction [69]. That said, improvements in clot burden appear to benefit from higher doses and longer infusion durations [69].

Future evidence may come from another currently enrolling RCT, HI-PEITHO (NCT04790370), which is comparing UACDT and anticoagulation in intermediate-high-risk PE [75].

### 10.3 Mechanical CDT

Mechanical CDTs for PE are based on thrombus fragmentation, fragmentation with aspiration, and rheolytic thrombectomy. Furthermore, mechanical approaches could be combined with the thrombolytic infusion into the residual clot. The main characteristics of the principal studies concerning mechanical CDT are described in Table 4.

#### 10.3.1 Pigtail Catheter

The simplest mechanical approach is represented by the rotatable pigtail catheter, which also allows thrombolytic infusion. This type of pigtail has an oval side hole on the outer curvature of the pigtail loop, allowing the passage of a guidewire, which facilitates manual rotation of the pigtail (Fig. 1, blue box) [76]. However, despite the lack of strong evidence, which is limited to case series, it surprisingly remains the most commonly used CDT for acute PE across Europe [39].

#### 10.3.2 BASHIR Endovascular Catheter 

The BASHIR^TM^ Endovascular Catheter (Thrombolex, Inc., New Britain, PA 18901, USA) features a spiral-cut infusion basket that can be collapsed and expanded repeatedly to create fissures in the clot, enabling both thrombolytic delivery and mechanical fragmentation of the thrombus (Fig. 1, blue box) [77]. This technology has demonstrated, in a prospective, multicenter study on intermediate-risk PE, a significant reduction of the RV/LV ratio by 33.3% at 48 hours and a 35.9% reduction in arterial obstruction, with minimal bleeding complications and device-related adverse events, showing a comparable hemodynamic benefit compared to other CDTs [59, 69, 70].

#### 10.3.3 Indigo System 

The Indigo^TM^ system (Penumbra, Inc., Alameda, California 94502, USA) consists of an 8-French aspiration catheter connected to a continuous suction vacuum system (Fig. 1, blue box). A wire separator within the catheter lumen aids in the retrieval of the clot. The catheter can be advanced through the thrombus multiple times to facilitate further clot removal. To minimize blood loss, it is essential to turn off the suction pump when the catheter is outside the thrombus [1, 67]. The newer generation of the Indigo system is equipped with a microprocessor to regulate aspiration, thereby minimizing blood loss [60]. The main advantage of this technology is the ability to avoid the use of thrombolytic agents.

The EXTRACT-PE trial, which enrolled 119 intermediate-risk PE patients treated with the Indigo system, demonstrated a good safety profile with a low rate of major adverse events (MAEs) (1.7%), a low bleeding rate at 48 hours (1.7%), and a low 30-day mortality rate (2.5%). Additionally, the trial showed a significant reduction in the RV/LV ratio by 27.3% at 48 hours and a reduction of sPAP by 7.9%. Furthermore, thrombolysis was avoided in 98.3% of procedures [61]. These results are supported by the interim analysis of the STRIKE-PE study, which shows a 25.7% reduction in RV/LV ratio at 48 hours and a 16.3% reduction in sPAP [60].

The ongoing prospective STORM-PE trial (NCT05684796), which compares anticoagulants alone versus anticoagulants plus the new-generation Indigo system, could help address the lack of data regarding the comparison with the current standard of care.

#### 10.3.4 FlowTriever

The FlowTriever^TM^ system (Inari Medical, Inc., Irvine, California 92618, USA) consists of a large-lumen aspiration catheter connected to a retraction aspirator (Fig. 1, blue box). The aspiration catheter is advanced into the thrombus to allow thrombus suction. If aspiration alone is insufficient, a flow restoration catheter can be introduced into the aspiration catheter. This flow restoration catheter consists of three self-expanding nitinol wire discs, which capture the thrombus and allow aspiration through the retraction of the catheter [67]. This technology also allows for the avoidance of thrombolytic agents.

As demonstrated in the prospective studies FLARE (intermediate-risk PE) and FLASH (intermediate-/high-risk PE), the FlowTriever system has shown a significant impact on hemodynamics, reducing the RV/LV ratio by 25–28% and sPAP by 22.2%. It also demonstrated a favorable safety profile, with MAEs occurring in 1.2%–3.8% of cases, major bleeding in 0.9%, and a low 30-day mortality [58, 62]. Interestingly, as reported in the FLAME study, in the high-risk PE population, FlowTriever appears to be associated with lower rates of mortality, major bleeding, and the need for bail-out therapy compared to other contemporary treatments, such as systemic thrombolysis and anticoagulation [71]. Furthermore, the recently published PEERLESS RCT, which enrolled 550 patients treated with either FlowTriever or CDTL, showed a significantly lower rate of clinical deterioration and/or bail-out procedures in the FlowTriever group. However, no differences in major bleeding or 30-day mortality were observed between the two groups [73].

#### 10.3.5 Cleaner 

The Cleaner^TM^ (Argon Medical, L.P., Fort Washington, Pennsylvania 19034, USA) consists of a catheter with a rotating tip that incorporates a flexible, spiral-shaped wire inside the catheter lumen (Fig. 1, blue box). The device is advanced through the thrombus under fluoroscopic guidance. The rotating, spiral wire at the tip of the catheter engages the thrombus by gently wrapping around and entangling the clot, allowing for effective disruption [78]. The use of the Cleaner in PE is limited to case series; however, the ongoing CLEAN-PE study (NCT06189313) aims to assess its safety and efficacy in patients with acute PE.

#### 10.3.6 AngioJet 

The AngioJet^TM^ catheter (Boston Scientific Corporation, Marlborough, Massachusetts 01752, USA) operates through a combination of thrombus fragmentation and aspiration (Fig. 1, blue box). Thrombus fragmentation is achieved by saline jets injected directly into the clot, while clot fragments are aspirated through the catheter’s side ports. The saline jets also facilitate the delivery of thrombolytic agents into the clot. Aspiration occurs via a Venturi effect, created by the high velocity of the saline injection. However, due to reported complications, including bradyarrhythmia, hemoglobinuria, renal insufficiency, hemoptysis, hemorrhages, and procedure-related deaths, the Food and Drug Administration (FDA) has issued a black box warning for the use of the AngioJet catheter in the pulmonary circulation [67, 79].

#### 10.3.7 PERT

As mentioned above, the management of PE requires prompt detection and rapid diagnosis, leading to timely selection and initiation of therapy tailored to the patient’s risk stratification and comorbidities, as well as close monitoring, particularly in intermediate-high and high-risk patients during the initial days. Therefore, effective management of PE necessitates the coordination of various specialists involved in the care of these patients. This need has led to the formation of Pulmonary Embolism Response Teams (PERT) in hospitals. PERT is a specialized, multidisciplinary group designed to provide rapid, coordinated care for patients with acute PE. The team typically includes pulmonologists, cardiologists, hematologists, intensivists/anesthetists, cardiothoracic surgeons, radiologists, and interventional specialists, all collaborating to deliver individualized care [10, 80]. The goal of PERT is to optimize patient outcomes by promptly assessing the severity of the PE and determining the most appropriate treatment strategy. The involvement of PERT has been associated with improved patient survival, reduced complications, and more efficient resource utilization, as it facilitates rapid decision-making and the timely implementation of therapeutic intervention [81, 82].

## 11. Integrating Current Recommendations and Emerging Evidence in Clinical Practice 

As mentioned above, current guidelines recommend the prompt initiation of anticoagulation and emerging reperfusion treatments, such as systemic thrombolysis, in high-risk patients, while anticoagulation alone is recommended for low- and intermediate-risk patients [10]. Despite these clear indications, systemic thrombolysis remains underused, with only 12–20% of high-risk PE patients receiving the treatment [2, 13, 14]. In contrast, nearly 40% of this population is reported to have contraindications to thrombolysis. According to the International Cooperative Pulmonary Embolism Registry (ICOPER), 28.9% of patients have had recent surgery, 11.2% have had recent trauma, 4.4% have low platelets, and 2.4% have active bleeding, making them ineligible for thrombolytic therapy [83]. On the other hand, the proportion of patients receiving advanced therapy is reported to be 14% in the low-risk group, 26% in the intermediate-low risk group, and 38% in the intermediate-high risk group [1]. Furthermore, a post hoc analysis of the PEITHO trial has shown that a subpopulation of patients with intermediate-high risk PE and higher-risk clinical features seems to benefit more from systemic thrombolysis than from anticoagulation alone [38]. Lastly, up to 8% of high-risk PE patients experience thrombolysis failure, and up to 5–6% of intermediate-high-risk patients experience hemodynamic decompensation and/or die within 72 hours of admission [14, 52].

These data highlight two major needs: first, the introduction of more accurate risk stratification tools to enable early detection of PE patients at higher risk of hemodynamic decompensation; and second, the availability of alternative advanced treatments.

Regarding the first, although not formally recommended, the use of the NEWS to predict 7-day intensive care unit admission, 30-day mortality, and the need for advanced therapy may help to identify early decompensation and select patients who could benefit from intensified treatment [41].

As for the second, CDT may represent an alternative treatment option for high-risk patients, for those with evidence of thrombolysis failure, or for intermediate–high risk patients at imminent risk of hemodynamic collapse. CDTs have demonstrated favorable hemodynamic effects, including a reduction in the RV/LV ratio by 25–38% and a decrease in sPAP by 7.9–22.2% [58, 59, 60, 61, 62]. They have shown a good safety profile, with a 30-day mortality rate of 0.9–2.7% in the intermediate-/high-risk population and a major bleeding rate ranging from 0.9% to 10% [58, 59, 60, 61, 62]. These rates are lower than those associated with contemporary therapies, such as systemic thrombolysis and anticoagulation, as reported in the FlowTriever for Acute Massive Pulmonary  Embolism (FLAME) study conducted on high-risk PE patients [71]. Importantly, even though it was not possible to perform a statistical evaluation of the differences between the two study arms, the FLAME study reported a very low in-hospital mortality rate of 1.9% in the FlowTriever group. In contrast, the historical mortality rate for high-risk PE was 28.5%. Compared to contemporary therapies, the FlowTriever strategy showed lower rates of bailout (3.8% vs. 26.2%), clinical deterioration (15.1% vs. 21.3%), and especially major bleeding (11.3% vs. 24.6%) [71]. While systemic thrombolytics remain the guideline-endorsed therapy and may be the only feasible option for patients too unstable to be transferred for alternative interventions, their routine use in high-risk cases warrants reconsideration given well-recognized limitations regarding both efficacy and safety. Mechanical CDTs could represent an effective alternative strategy even in high-risk patients, particularly in those with an elevated bleeding risk. Despite these encouraging results for CDTs, significant limitations remain due to the lack of large randomized studies directly comparing them with current standard therapies. Ongoing trials, such as the STORM-PE trial (NCT05684796), which compares anticoagulants alone versus anticoagulants plus the new-generation Indigo system; the CLEAN-PE study (NCT06189313), which aims to assess the safety and efficacy of the Cleaner system in patients with acute PE; the BETULA RCT (NCT03854266), which includes intermediate–high-risk PE patients treated with the Uni-Fuse system or heparin alone; and the PE-TRACT trial (NCT05591118), comparing CDT or mechanical thrombectomy plus anticoagulation versus anticoagulation alone in intermediate–high-risk PE, could help address the current lack of comparative data with standard care.

Moreover, as mentioned earlier, a post-hoc analysis of the PEITHO trial identified a subgroup of intermediate-risk PE patients with a high likelihood of hemodynamic collapse, who may benefit from more intensive treatments such as thrombolysis, albeit at the non-negligible cost of an increased incidence of major bleeding and stroke [38]. A currently ongoing RCT, HI-PEITHO (NCT04790370), which is comparing ultrasound-assisted CDT with anticoagulation in intermediate–high-risk PE, may help clarify the optimal treatment strategy in this specific population [75].

**Fig. 2.**
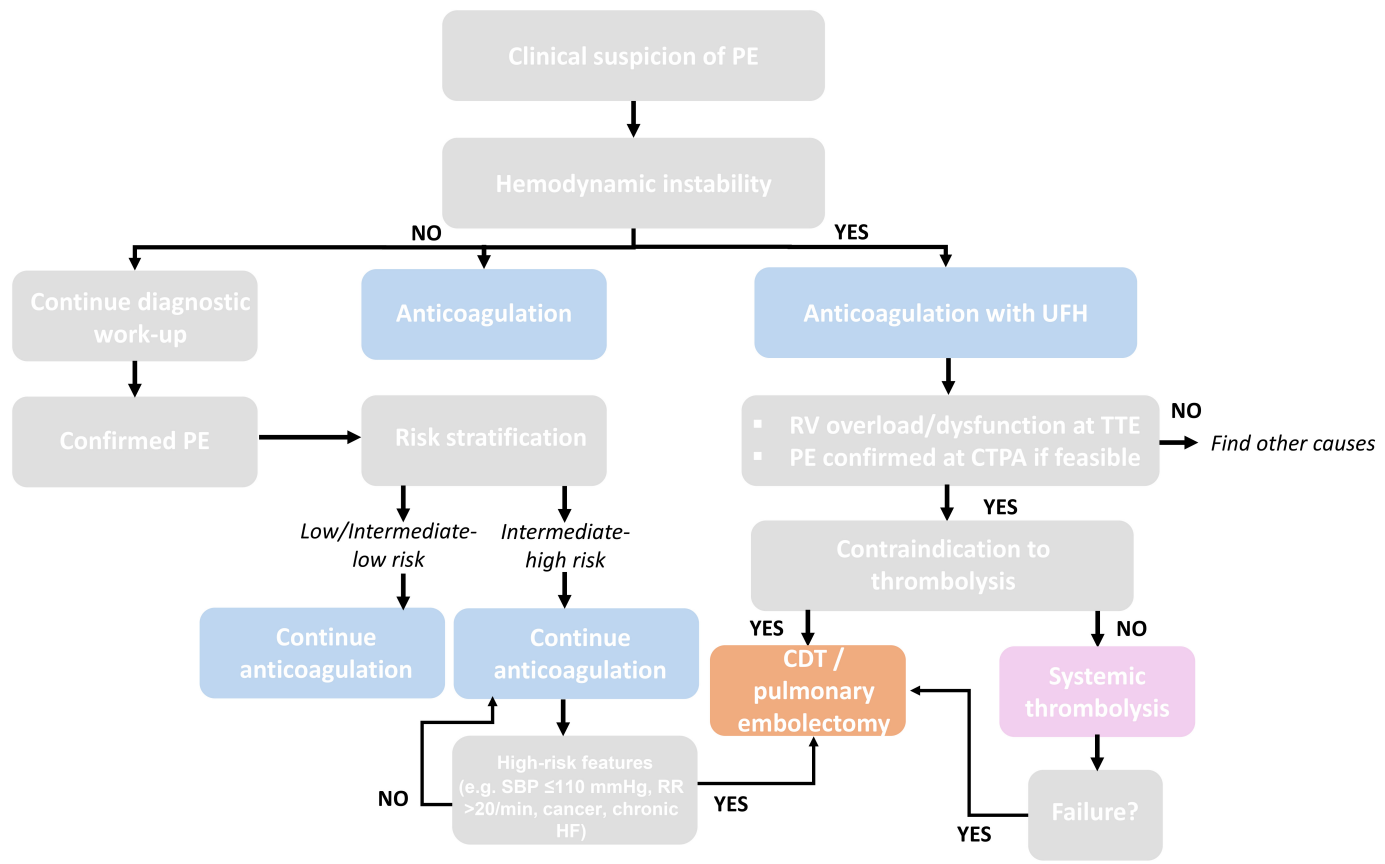
**Proposed algorithms for the management of PE**. Flowchart illustrating the diagnostic and therapeutic approach to patients with suspected pulmonary embolism according to hemodynamic stability and risk stratification [10, 38, 71]. CDT, catheter-directed therapies; CTPA, computed tomography pulmonary angiography; HF, heart failure; RR, respiratory rate; RV, right ventricle; SBP, systolic blood pressure; TTE, transthoracic echocardiogram.

Lastly, mechanical CDTs provide a reasonable alternative for all patients with contraindications to thrombolysis or those at high risk for bleeding, avoiding thrombolytic agent administration. In Fig. 2 (Ref. [10, 38, 71]), we propose a flowchart—developed on the basis of current guideline recommendations and recent evidence—illustrating the diagnostic and therapeutic approaches to patients with suspected PE according to hemodynamic stability and risk stratification. Initial management includes anticoagulation, with UFH preferred in unstable patients. In high-risk cases, systemic thrombolysis is recommended when not contraindicated, while CDT or surgical embolectomy represent alternatives for patients with contraindications to thrombolysis or after treatment failure. In intermediate-high-risk patients with features suggestive of impending hemodynamic collapse, advanced treatment strategies such as CDT or surgical embolectomy may be considered, depending on institutional expertise and postoperative management capabilities [10, 38, 71].

Ultimately, particular attention should be given to machine learning (ML) models, which are increasingly being developed in the field of PE. By leveraging clinical data to capture high-dimensional and nonlinear relationships among patient features, ML can accurately forecast clinical outcomes. Owing to their strong learning capabilities and predictive performance, ML-based approaches may support clinical decision-making with greater accuracy than traditional statistical methods. One ML model has been designed to identify patients at risk of PE even before its onset, thereby offering the possibility of earlier recognition, diagnosis, and timely treatment [84]. Similarly, ML has been applied to identify predictors of adverse outcomes, which may help stratify patients who could benefit from more intensive treatment prior to hemodynamic deterioration. In this context, an ML model developed for patients with central PE identified elevated sPESI scores, leucocytosis, increased serum creatinine, elevated troponin levels, and higher respiratory rates as independent predictors of adverse outcomes [85]. Furthermore, another ML model has been validated to predict 30-day mortality in critically ill patients with concomitant PE and HF in the intensive care unit setting [86]. Lastly, ML models can also be applied during the diagnostic phase on CT studies and have been shown to detect PE with high sensitivity and specificity, even in scans not specifically performed for PE evaluation [87]. This approach may prove useful in improving both the accuracy and the speed of PE detection. Collectively, these tools have the potential to improve the management of PE by enabling timely diagnosis and supporting more tailored therapeutic strategies.

## 12. Conclusion

PE continues to be the third leading cause of cardiovascular death, with high mortality rates within 30 days. Its rising incidence over the last few decades has highlighted the urgent need to improve both management and treatment strategies. While current guidelines clearly recommend the prompt initiation of anticoagulation and systemic thrombolysis in high-risk patients, and anticoagulation alone for low- and intermediate-risk patients, data from registries show that the PE population is highly heterogeneous, and the majority of patients cannot be treated with the standard of care. In recent years, emerging therapies have gained substantial evidence, demonstrating an excellent safety profile, lower bleeding rates, and reduced mortality. These therapies also have a beneficial impact on hemodynamics, making them suitable for patients with contraindications to thrombolysis or those at a higher bleeding risk.

## Abbreviations

CDT, catheter-directed therapy; CDTL, catheter-directed thrombolysis; CTPA, computed tomography pulmonary angiography; CUS, lower-limb compression ultrasonography; DVT, deep vein thrombosis; HF, heart failure; LMWH, low-molecular-weight heparin; ML, machine learning; NOAC, novel oral anticoagulant; PE, pulmonary embolism; PESI, pulmonary embolism severity index; RV, right ventricle; sPAP, systolic pulmonary artery pressure; TTE, transthoracic echocardiogram; UACTD, ultrasound-assisted catheter-directed therapy; UFH, unfractionated heparin; VKA, vitamin K antagonists; VTE, venous thromboembolism.

## References

[1] Schultz J, Giordano N, Zheng H, Parry BA, Barnes GD, Heresi GA (2019). EXPRESS: A Multidisciplinary Pulmonary Embolism Response Team (PERT) - Experience from a national multicenter consortium. *Pulmonary Circulation*.

[2] Jiménez D, de Miguel-Díez J, Guijarro R, Trujillo-Santos J, Otero R, Barba R (2016). Trends in the Management and Outcomes of Acute Pulmonary Embolism: Analysis From the RIETE Registry. *Journal of the American College of Cardiology*.

[3] Wendelboe AM, Raskob GE (2016). Global Burden of Thrombosis: Epidemiologic Aspects. *Circulation Research*.

[4] Huisman MV, Barco S, Cannegieter SC, Le Gal G, Konstantinides SV, Reitsma PH (2018). Pulmonary embolism. *Nature Reviews. Disease Primers*.

[5] Silver MJ, Giri J, Duffy Á, Jaber WA, Khandhar S, Ouriel K (2023). Incidence of Mortality and Complications in High-Risk Pulmonary Embolism: A Systematic Review and Meta-Analysis. *Journal of the Society for Cardiovascular Angiography & Interventions*.

[6] Temgoua MN, Tochie JN, Noubiap JJ, Agbor VN, Danwang C, Endomba FTA (2017). Global incidence and case fatality rate of pulmonary embolism following major surgery: a protocol for a systematic review and meta-analysis of cohort studies. *Systematic Reviews*.

[7] Caron A, Depas N, Chazard E, Yelnik C, Jeanpierre E, Paris C (2019). Risk of Pulmonary Embolism More Than 6 Weeks After Surgery Among Cancer-Free Middle-aged Patients. *JAMA Surgery*.

[8] Gregson J, Kaptoge S, Bolton T, Pennells L, Willeit P, Burgess S (2019). Cardiovascular Risk Factors Associated With Venous Thromboembolism. *JAMA Cardiology*.

[9] GBD 2021 Adult BMI Collaborators (2025). Global, regional, and national prevalence of adult overweight and obesity, 1990-2021, with forecasts to 2050: a forecasting study for the Global Burden of Disease Study 2021. *Lancet (London, England)*.

[10] Konstantinides SV, Meyer G, Becattini C, Bueno H, Geersing GJ, Harjola VP (2020). 2019 ESC Guidelines for the diagnosis and management of acute pulmonary embolism developed in collaboration with the European Respiratory Society (ERS). *European Heart Journal*.

[11] Marti C, John G, Konstantinides S, Combescure C, Sanchez O, Lankeit M (2015). Systemic thrombolytic therapy for acute pulmonary embolism: a systematic review and meta-analysis. *European Heart Journal*.

[12] Chatterjee S, Chakraborty A, Weinberg I, Kadakia M, Wilensky RL, Sardar P (2014). Thrombolysis for pulmonary embolism and risk of all-cause mortality, major bleeding, and intracranial hemorrhage: a meta-analysis. *JAMA*.

[13] Keller K, Hobohm L, Ebner M, Kresoja KP, Münzel T, Konstantinides SV (2020). Trends in thrombolytic treatment and outcomes of acute pulmonary embolism in Germany. *European Heart Journal*.

[14] Kobayashi T, Pugliese S, Sethi SS, Parikh SA, Goldberg J, Alkhafan F (2024). Contemporary Management and Outcomes of Patients With High-Risk Pulmonary Embolism. *Journal of the American College of Cardiology*.

[15] Lehnert P, Lange T, Møller CH, Olsen PS, Carlsen J (2018). Acute Pulmonary Embolism in a National Danish Cohort: Increasing Incidence and Decreasing Mortality. *Thrombosis and Haemostasis*.

[16] Zhen K, Tao Y, Xia L, Wang S, Gao Q, Wang D (2025). Epidemiology of pulmonary embolism in China, 2021: a nationwide hospital-based study. *The Lancet Regional Health. Western Pacific*.

[17] Lutsey PL, Zakai NA (2023). Epidemiology and prevention of venous thromboembolism. *Nature Reviews. Cardiology*.

[18] Cohen AT, Agnelli G, Anderson FA, Arcelus JI, Bergqvist D, Brecht JG (2007). Venous thromboembolism (VTE) in Europe. The number of VTE events and associated morbidity and mortality. *Thrombosis and Haemostasis*.

[19] Anderson FA, Spencer FA (2003). Risk factors for venous thromboembolism. *Circulation*.

[20] Rogers MAM, Levine DA, Blumberg N, Flanders SA, Chopra V, Langa KM (2012). Triggers of hospitalization for venous thromboembolism. *Circulation*.

[21] White RH (2003). The epidemiology of venous thromboembolism. *Circulation*.

[22] Pollack CV, Schreiber D, Goldhaber SZ, Slattery D, Fanikos J, O’Neil BJ (2011). Clinical characteristics, management, and outcomes of patients diagnosed with acute pulmonary embolism in the emergency department: initial report of EMPEROR (Multicenter Emergency Medicine Pulmonary Embolism in the Real World Registry). *Journal of the American College of Cardiology*.

[23] Miniati M, Prediletto R, Formichi B, Marini C, Di Ricco G, Tonelli L (1999). Accuracy of clinical assessment in the diagnosis of pulmonary embolism. *American Journal of Respiratory and Critical Care Medicine*.

[24] Wells PS, Ginsberg JS, Anderson DR, Kearon C, Gent M, Turpie AG (1998). Use of a clinical model for safe management of patients with suspected pulmonary embolism. *Annals of Internal Medicine*.

[25] Prandoni P, Lensing AWA, Prins MH, Ciammaichella M, Perlati M, Mumoli N (2016). Prevalence of Pulmonary Embolism among Patients Hospitalized for Syncope. *The New England Journal of Medicine*.

[26] Santolicandro A, Prediletto R, Fornai E, Formichi B, Begliomini E, Giannella-Neto A (1995). Mechanisms of hypoxemia and hypocapnia in pulmonary embolism. *American Journal of Respiratory and Critical Care Medicine*.

[27] Klok FA, Mos ICM, Nijkeuter M, Righini M, Perrier A, Le Gal G (2008). Simplification of the revised Geneva score for assessing clinical probability of pulmonary embolism. *Archives of Internal Medicine*.

[28] Wells PS, Anderson DR, Rodger M, Stiell I, Dreyer JF, Barnes D (2001). Excluding pulmonary embolism at the bedside without diagnostic imaging: management of patients with suspected pulmonary embolism presenting to the emergency department by using a simple clinical model and d-dimer. *Annals of Internal Medicine*.

[29] Di Nisio M, Squizzato A, Rutjes AWS, Büller HR, Zwinderman AH, Bossuyt PMM (2007). Diagnostic accuracy of D-dimer test for exclusion of venous thromboembolism: a systematic review. *Journal of Thrombosis and Haemostasis: JTH*.

[30] Le Gal G, Righini M, Sanchez O, Roy PM, Baba-Ahmed M, Perrier A (2006). A positive compression ultrasonography of the lower limb veins is highly predictive of pulmonary embolism on computed tomography in suspected patients. *Thrombosis and Haemostasis*.

[31] Dresden S, Mitchell P, Rahimi L, Leo M, Rubin-Smith J, Bibi S (2014). Right ventricular dilatation on bedside echocardiography performed by emergency physicians aids in the diagnosis of pulmonary embolism. *Annals of Emergency Medicine*.

[32] Goldberg JB, Giri J, Kobayashi T, Ruel M, Mittnacht AJC, Rivera-Lebron B (2023). Surgical Management and Mechanical Circulatory Support in High-Risk Pulmonary Embolisms: Historical Context, Current Status, and Future Directions: A Scientific Statement From the American Heart Association. *Circulation*.

[33] Calé R, Ascenção R, Bulhosa C, Pereira H, Borges M, Costa J (2025). In-hospital mortality of high-risk pulmonary embolism: a nationwide population-based cohort study in Portugal from 2010 to 2018. *Pulmonology*.

[34] Schoepf UJ, Kucher N, Kipfmueller F, Quiroz R, Costello P, Goldhaber SZ (2004). Right ventricular enlargement on chest computed tomography: a predictor of early death in acute pulmonary embolism. *Circulation*.

[35] Meinel FG, Nance JW, Schoepf UJ, Hoffmann VS, Thierfelder KM, Costello P (2015). Predictive Value of Computed Tomography in Acute Pulmonary Embolism: Systematic Review and Meta-analysis. *The American Journal of Medicine*.

[36] Becattini C, Vedovati MC, Agnelli G (2007). Prognostic value of troponins in acute pulmonary embolism: a meta-analysis. *Circulation*.

[37] Keeling WB, Sundt T, Leacche M, Okita Y, Binongo J, Lasajanak Y (2016). Outcomes After Surgical Pulmonary Embolectomy for Acute Pulmonary Embolus: A Multi-Institutional Study. *The Annals of Thoracic Surgery*.

[38] Barco S, Vicaut E, Klok FA, Lankeit M, Meyer G, Konstantinides SV (2018). Improved identification of thrombolysis candidates amongst intermediate-risk pulmonary embolism patients: implications for future trials. *The European Respiratory Journal*.

[39] Pruszczyk P, Klok FA, Kucher N, Roik M, Meneveau N, Sharp ASP (2022). Percutaneous treatment options for acute pulmonary embolism: a clinical consensus statement by the ESC Working Group on Pulmonary Circulation and Right Ventricular Function and the European Association of Percutaneous Cardiovascular Interventions. *EuroIntervention: Journal of EuroPCR in Collaboration with the Working Group on Interventional Cardiology of the European Society of Cardiology*.

[40] Bavalia R, Stals MAM, Mulder FI, Bistervels IM, Coppens M, Faber LM (2023). Use of the National Early Warning Score for predicting deterioration of patients with acute pulmonary embolism: a post-hoc analysis of the YEARS Study. *Emergency Medicine Journal: EMJ*.

[41] Janata K, Lipa AJ, Merrelaar A, Merrelaar M, Azizi-Semrad U, Herkner H (2025). Enhancing Pulmonary Embolism Risk Stratification: The National Early Warning Score and Its Integration into the European Society of Cardiology Classification. *Thrombosis and Haemostasis*.

[42] Giraud R, Laurencet M, Assouline B, De Charrière A, Banfi C, Bendjelid K (2021). Can VA-ECMO Be Used as an Adequate Treatment in Massive Pulmonary Embolism?. *Journal of Clinical Medicine*.

[43] Banfi C, Assouline B, Bendjelid K, Giraud R (2022). Can a Heart Recently Recovered from an Acute Pulmonary Embolism Supported by Venoarterial Extracorporeal Membrane Oxygenation be Considered for Donation?. *ASAIO Journal (American Society for Artificial Internal Organs: 1992)*.

[44] Pavlovic G, Banfi C, Tassaux D, Peter RE, Licker MJ, Bendjelid K (2014). Peri-operative massive pulmonary embolism management: is veno-arterial ECMO a therapeutic option?. *Acta Anaesthesiologica Scandinavica*.

[45] Corsi F, Lebreton G, Bréchot N, Hekimian G, Nieszkowska A, Trouillet JL (2017). Life-threatening massive pulmonary embolism rescued by venoarterial-extracorporeal membrane oxygenation. *Critical Care (London, England)*.

[46] Boey JJE, Dhundi U, Ling RR, Chiew JK, Fong NCJ, Chen Y (2023). Extracorporeal Membrane Oxygenation for Pulmonary Embolism: A Systematic Review and Meta-Analysis. *Journal of Clinical Medicine*.

[47] Assouline B, Assouline-Reinmann M, Giraud R, Levy D, Saura O, Bendjelid K (2022). Management of High-Risk Pulmonary Embolism: What Is the Place of Extracorporeal Membrane Oxygenation?. *Journal of Clinical Medicine*.

[48] Giraud R, Banfi C, Siegenthaler N, Bendjelid K (2016). Massive pulmonary embolism leading to cardiac arrest: one pathology, two different ECMO modes to assist patients. *Journal of Clinical Monitoring and Computing*.

[49] EINSTEIN–PE Investigators, Büller HR, Prins MH, Lensin AWA, Decousus H, Jacobson BF (2012). Oral rivaroxaban for the treatment of symptomatic pulmonary embolism. *The New England Journal of Medicine*.

[50] Agnelli G, Buller HR, Cohen A, Curto M, Gallus AS, Johnson M (2013). Oral apixaban for the treatment of acute venous thromboembolism. *The New England Journal of Medicine*.

[51] van der Hulle T, Kooiman J, den Exter PL, Dekkers OM, Klok FA, Huisman MV (2014). Effectiveness and safety of novel oral anticoagulants as compared with vitamin K antagonists in the treatment of acute symptomatic venous thromboembolism: a systematic review and meta-analysis. *Journal of Thrombosis and Haemostasis: JTH*.

[52] Meyer G, Vicaut E, Danays T, Agnelli G, Becattini C, Beyer-Westendorf J (2014). Fibrinolysis for patients with intermediate-risk pulmonary embolism. *The New England Journal of Medicine*.

[53] Samama CM, Afshari A, Grønlykke L, Madsen MH, Wiberg S, Romero CS (2024). European guidelines on peri-operative venous thromboembolism prophylaxis: first update.: Executive summary. *European Journal of Anaesthesiology*.

[54] Douketis JD, Spyropoulos AC, Murad MH, Arcelus JI, Dager WE, Dunn AS (2022). Perioperative Management of Antithrombotic Therapy: An American College of Chest Physicians Clinical Practice Guideline. *Chest*.

[55] Abraham P, Arroyo DA, Giraud R, Bounameaux H, Bendjelid K (2018). Understanding haemorrhagic risk following thrombolytic therapy in patients with intermediate-risk and high-risk pulmonary embolism: a hypothesis paper. *Open Heart*.

[56] Jerjes-Sanchez C, Ramírez-Rivera A, de Lourdes García M, Arriaga-Nava R, Valencia S, Rosado-Buzzo A (1995). Streptokinase and Heparin versus Heparin Alone in Massive Pulmonary Embolism: A Randomized Controlled Trial. *Journal of Thrombosis and Thrombolysis*.

[57] Poterucha TJ, Bergmark B, Aranki S, Kaneko T, Piazza G (2015). Surgical Pulmonary Embolectomy. *Circulation*.

[58] Toma C, Jaber WA, Weinberg MD, Bunte MC, Khandhar S, Stegman B (2023). Acute outcomes for the full US cohort of the FLASH mechanical thrombectomy registry in pulmonary embolism. *EuroIntervention: Journal of EuroPCR in Collaboration with the Working Group on Interventional Cardiology of the European Society of Cardiology*.

[59] Bashir R, Foster M, Iskander A, Darki A, Jaber W, Rali PM (2022). Pharmacomechanical Catheter-Directed Thrombolysis With the Bashir Endovascular Catheter for Acute Pulmonary Embolism: The RESCUE Study. *JACC. Cardiovascular Interventions*.

[60] Moriarty JM, Dohad SY, Schiro BJ, Tamaddon H, Heithaus RE, Iliadis EA (2024). Clinical, Functional, and Quality-of-Life Outcomes after Computer Assisted Vacuum Thrombectomy for Pulmonary Embolism: Interim Analysis of the STRIKE-PE Study. *Journal of Vascular and Interventional Radiology: JVIR*.

[61] Sista AK, Horowitz JM, Tapson VF, Rosenberg M, Elder MD, Schiro BJ (2021). Indigo Aspiration System for Treatment of Pulmonary Embolism: Results of the EXTRACT-PE Trial. *JACC. Cardiovascular Interventions*.

[62] Tu T, Toma C, Tapson VF, Adams C, Jaber WA, Silver M (2019). A Prospective, Single-Arm, Multicenter Trial of Catheter-Directed Mechanical Thrombectomy for Intermediate-Risk Acute Pulmonary Embolism: The FLARE Study. *JACC. Cardiovascular Interventions*.

[63] Costa F, Salinas P, Iannaccone M, Cerrato E, Márquez DT, Misra S (2025). Catheter-based techniques for pulmonary embolism treatment. *EuroIntervention*.

[64] Sadeghipour P, Jenab Y, Moosavi J, Hosseini K, Mohebbi B, Hosseinsabet A (2022). Catheter-Directed Thrombolysis vs Anticoagulation in Patients With Acute Intermediate-High-risk Pulmonary Embolism: The CANARY Randomized Clinical Trial. *JAMA Cardiology*.

[65] Kucher N, Boekstegers P, Müller OJ, Kupatt C, Beyer-Westendorf J, Heitzer T (2014). Randomized, controlled trial of ultrasound-assisted catheter-directed thrombolysis for acute intermediate-risk pulmonary embolism. *Circulation*.

[66] Kroupa J, Buk M, Weichet J, Malikova H, Bartova L, Linkova H (2022). A pilot randomised trial of catheter-directed thrombolysis or standard anticoagulation for patients with intermediate-high risk acute pulmonary embolism. *EuroIntervention: Journal of EuroPCR in Collaboration with the Working Group on Interventional Cardiology of the European Society of Cardiology*.

[67] Nosher JL, Patel A, Jagpal S, Gribbin C, Gendel V (2017). Endovascular treatment of pulmonary embolism: Selective review of available techniques. *World Journal of Radiology*.

[68] Piazza G, Hohlfelder B, Jaff MR, Ouriel K, Engelhardt TC, Sterling KM (2015). A Prospective, Single-Arm, Multicenter Trial of Ultrasound-Facilitated, Catheter-Directed, Low-Dose Fibrinolysis for Acute Massive and Submassive Pulmonary Embolism: The SEATTLE II Study. *JACC. Cardiovascular Interventions*.

[69] Tapson VF, Sterling K, Jones N, Elder M, Tripathy U, Brower J (2018). A Randomized Trial of the Optimum Duration of Acoustic Pulse Thrombolysis Procedure in Acute Intermediate-Risk Pulmonary Embolism: The OPTALYSE PE Trial. *JACC. Cardiovascular Interventions*.

[70] Sterling KM, Goldhaber SZ, Sharp ASP, Kucher N, Jones N, Maholic R (2024). Prospective Multicenter International Registry of Ultrasound-Facilitated Catheter-Directed Thrombolysis in Intermediate-High and High-Risk Pulmonary Embolism (KNOCOUT PE). *Circulation. Cardiovascular Interventions*.

[71] Silver MJ, Gibson CM, Giri J, Khandhar S, Jaber W, Toma C (2023). Outcomes in High-Risk Pulmonary Embolism Patients Undergoing FlowTriever Mechanical Thrombectomy or Other Contemporary Therapies: Results From the FLAME Study. *Circulation. Cardiovascular Interventions*.

[72] Avgerinos ED, Jaber W, Lacomis J, Markel K, McDaniel M, Rivera-Lebron BN (2021). Randomized Trial Comparing Standard Versus Ultrasound-Assisted Thrombolysis for Submassive Pulmonary Embolism: The SUNSET sPE Trial. *JACC. Cardiovascular Interventions*.

[73] Jaber WA, Gonsalves CF, Stortecky S, Horr S, Pappas O, Gandhi RT (2025). Large-Bore Mechanical Thrombectomy Versus Catheter-Directed Thrombolysis in the Management of Intermediate-Risk Pulmonary Embolism: Primary Results of the PEERLESS Randomized Controlled Trial. *Circulation*.

[74] Planer D, Yanko S, Matok I, Paltiel O, Zmiro R, Rotshild V (2023). Catheter-directed thrombolysis compared with systemic thrombolysis and anticoagulation in patients with intermediate- or high-risk pulmonary embolism: systematic review and network meta-analysis. *CMAJ: Canadian Medical Association Journal = Journal De L’Association Medicale Canadienne*.

[75] Klok FA, Piazza G, Sharp ASP, Ní Ainle F, Jaff MR, Chauhan N (2022). Ultrasound-facilitated, catheter-directed thrombolysis vs anticoagulation alone for acute intermediate-high-risk pulmonary embolism: Rationale and design of the HI-PEITHO study. *American Heart Journal*.

[76] Schmitz-Rode T, Janssens U, Duda SH, Erley CM, Günther RW (2000). Massive pulmonary embolism: percutaneous emergency treatment by pigtail rotation catheter. *Journal of the American College of Cardiology*.

[77] Singh M, Quimby A, Lakhter V, Al-Otaibi M, Rali PM, Bashir R (2021). Novel Pharmacomechanical Thrombolysis for Treating Intermediate-Risk Acute Pulmonary Embolism: The Bashir Endovascular Catheter. *Texas Heart Institute Journal*.

[78] Barjaktarevic I, Friedman O, Ishak C, Sista AK (2014). Catheter-directed clot fragmentation using the Cleaner™ device in a patient presenting with massive pulmonary embolism. *Journal of Radiology Case Reports*.

[79] Bonvini RF, Roffi M, Bounameaux H, Noble S, Müller H, Keller PF (2013). AngioJet rheolytic thrombectomy in patients presenting with high-risk pulmonary embolism and cardiogenic shock: a feasibility pilot study. *EuroIntervention: Journal of EuroPCR in Collaboration with the Working Group on Interventional Cardiology of the European Society of Cardiology*.

[80] Kabrhel C, Rosovsky R, Channick R, Jaff MR, Weinberg I, Sundt T (2016). A Multidisciplinary Pulmonary Embolism Response Team: Initial 30-Month Experience With a Novel Approach to Delivery of Care to Patients With Submassive and Massive Pulmonary Embolism. *Chest*.

[81] Jaff MR, Secemsky EA (2021). Response Team Management of Acute Serious Pulmonary Embolism. *Texas Heart Institute Journal*.

[82] Porres-Aguilar M, Rosovsky RP, Rivera-Lebron BN, Kaatz S, Mukherjee D, Anaya-Ayala JE (2022). Pulmonary embolism response teams: Changing the paradigm in the care for acute pulmonary embolism. *Journal of Thrombosis and Haemostasis: JTH*.

[83] Kucher N, Rossi E, De Rosa M, Goldhaber SZ (2006). Massive pulmonary embolism. *Circulation*.

[84] Ryan L, Maharjan J, Mataraso S, Barnes G, Hoffman J, Mao Q (2022). Predicting pulmonary embolism among hospitalized patients with machine learning algorithms. *Pulmonary Circulation*.

[85] Cantu-Martinez O, Martinez Manzano JM, Tito S, Prendergast A, Jarrett SA, Chiang B (2023). Clinical features and risk factors of adverse clinical outcomes in central pulmonary embolism using machine learning analysis. *Respiratory Medicine*.

[86] Liu J, Li R, Yao T, Liu G, Guo L, He J (2024). Interpretable Machine Learning Approach for Predicting 30-Day Mortality of Critical Ill Patients with Pulmonary Embolism and Heart Failure: A Retrospective Study. *Clinical and Applied Thrombosis/hemostasis: Official Journal of the International Academy of Clinical and Applied Thrombosis/Hemostasis*.

[87] Farzaneh H, Junn J, Chaibi Y, Ayobi A, Franciosini A, Scudeler M (2025). Deep Learning-Based Algorithm for Automatic Detection of Incidental Pulmonary Embolism on Contrast-Enhanced Computed Tomography: A Multicenter Multivendor Study. *Radiology Advances*.

